# Optimization of linezolid infusion duration in critically ill patients: a population pharmacokinetic analysis of the trade-off between efficacy and toxicity

**DOI:** 10.3389/fphar.2026.1766346

**Published:** 2026-06-30

**Authors:** Chenchen Wu, Jiaqun Wang, Weihua Sun, Wenjun Zhao, Wendi Zhao, Qingqing Yang, Li Xue, Lingti Kong

**Affiliations:** 1 Department of Endocrine and Metabolic Diseases, The First Affiliated Hospital of Bengbu Medical University, Bengbu, Anhui, China; 2 School of Computer and Information Engineering, Anhui University of Finance and Economics, Bengbu, Anhui, China; 3 Anhui Xiangyuan Technology Co., Ltd., Bengbu, Anhui, China; 4 Emergency Medicine, The Third The People’s Hospital of Bengbu, Bengbu, Anhui, China; 5 Department of Pharmacy, The First Affiliated Hospital of Bengbu Medical University, Bengbu, Anhui, China

**Keywords:** composite efficacy-safety target, infusion duration, linezolid, Monte Carlo simulation, population pharmacokinetics

## Abstract

**Background:**

Linezolid is a cornerstone therapy for multidrug-resistant Gram-positive infections. While prolonged intermittent infusion (PII) or continuous infusion (CI) can optimize the efficacy of time-dependent antibiotics, their application to linezolid remains controversial. Specifically, it is debated whether the theoretical efficacy gains of CI are negated by the elimination of the concentration nadir, which may trigger accumulation-related toxicity. Quantitative evidence dissecting this delicate trade-off is currently absent.

**Objectives:**

This study aimed to quantify the interplay between infusion duration, efficacy, and toxicity. By developing a population pharmacokinetic (PopPK) model and employing a “composite efficacy-safety target”, we sought to identify precision dosing strategies for critically ill patients.

**Methods:**

Plasma concentration-time data obtained from 20 critically ill patients were used to develop a PopPK model using nonlinear mixed-effects modeling (NONMEM v7.5). Based on final model parameters, Monte Carlo simulations were performed for 1,000 virtual patients. A composite target was established comprising a pharmacodynamic efficacy threshold (AUC_24_,ss/minimum inhibitory concentration ≥100 and %T > MIC ≥85%) and a safety threshold (steady-state trough concentration, C_min,ss_ < 8 mg/L). Probability of Target Attainment (PTA) was evaluated across 7 dosing regimens (300, … , 900 mg q12 h) and 13 infusion durations (0.5, 1, 2, … , 12 h).

**Results:**

The pharmacokinetics were best described by a one-compartment model, with body weight (WT) and creatinine clearance (CLcr) identified as critical determinants of distribution volume (V) and clearance (CL), respectively. Simulations revealed a non-linear relationship between infusion duration and composite PTA. While prolonged intermittent infusion enhanced efficacy by improving % T > MIC, indiscriminate extension to CI, particularly at moderate-to-high doses, resulted in a systematic breach of the safety threshold (C_min,ss_ ≥ 8 mg/L). Consequently, composite PTA declined due to toxic over-exposure. Notably, for refractory pathogens (MIC ≥2 mg/L), traditional CI yielded near-zero success rates due to high toxicity risks.

**Conclusion:**

In critically ill patients, the optimal infusion duration for linezolid exhibits a non-linear dependency on dose and MIC. For high-MIC pathogens requiring intensified dosing (e.g., 900 mg q12 h), a prolonged infusion of 2–5 h optimizes the therapeutic index, synergizing maximal efficacy with a necessary elimination phase to mitigate toxicity.

## 1 Introduction

Linezolid, a widely used oxazolidinone antibiotic, inhibits bacterial protein synthesis. This unique mechanism confers a lack of cross-resistance with traditional cell walls or DNA-targeting agents, maintaining robust activity against multidrug-resistant (MDR) pathogens such as methicillin-resistant *Staphylococcus aureus* (MRSA) and vancomycin-resistant enterococci (VRE) (Long and Birte, 2012; Belousoff et al., 2017). Additionally, its high oral bioavailability and tissue penetration, achieving epithelial lining fluid concentrations equal to or exceeding plasma levels, make it an important therapeutic option for hospital-acquired and ventilator-associated pneumonia (HAP/VAP) (Boselli et al., 2005; De Pascale et al., 2015). Clinical trials have shown that linezolid is non-inferior and, in some studies, superior to vancomycin for the treatment of MRSA pneumonia (Wunderink et al., 2012; Kato et al., 2021; Kalil et al., 2016; Saukkonen et al., 2025). However, critically ill patients frequently exhibit profound pharmacokinetic alterations and substantial interpatient variability (Roger et al., 2025), which may complicate the attainment of optimal linezolid exposure and highlight the need for dosing optimization strategies in this population. Pathophysiological changes such as systemic inflammatory response syndrome (SIRS) and aggressive fluid resuscitation may increase the volume of distribution through capillary leakage (Roberts and Jeffrey, 2009). At the same time, renal function may fluctuate markedly, ranging from acute kidney injury (AKI) (Sazdanovic et al., 2016) to augmented renal clearance (ARC) (Gorham, Taccone, and Hites, 2022). These dynamic changes may lead to either subtherapeutic exposure or excessive drug accumulation under standard dosing regimens, thereby complicating the achievement of optimal therapeutic exposure.

For linezolid, pharmacokinetic–pharmacodynamic (PK/PD) targets are essential for balancing efficacy and safety. Antibacterial efficacy is primarily associated with the area under the concentration–time curve (AUC) to minimum inhibitory concentration (MIC) ratio (AUC_24_/MIC, target 80–120) and the percentage of time above MIC (%T > MIC ≥85%) (Abdul-Aziz et al., 2020; Heidari and Khalili, 2023). However, toxicity is also a critical concern. Linezolid-induced myelosuppression, particularly thrombocytopenia, has been associated with excessive drug exposure (Liu H. X. et al., 2024). Although debate persists regarding the most predictive exposure metric (Zoller et al., 2022), accumulating evidence suggests that the steady-state trough concentration (C_min,ss_) is a practical clinical indicator of toxicity risk (Matsumoto et al., 2014; Boak et al., 2014; Bandín-Vilar et al., 2022). Specifically, when C_min,ss_ exceeds 8 mg/L, the risk of thrombocytopenia increases substantially, and clinical recommendations generally advise maintaining trough concentrations below this threshold.

The need to achieve adequate antibacterial exposure while avoiding toxic accumulation creates a significant therapeutic dilemma. Strategies commonly used for *β*-lactam antibiotics, such as extending infusion duration, have been proposed to improve pharmacodynamic target attainment by increasing %T > MIC(Zhao et al., 2021; Hui et al., 2022; Hui et al., 2024; Barrasa, Valdazo, and Martín, 2025). However, the application of this paradigm to linezolid remains controversial. Unlike *β*-lactams, linezolid toxicity is exposure-related, and prolonged or continuous infusion may increase the PTA (Zhao et al., 2021; Zavřelová et al., 2025). From a pharmacokinetic perspective, extending infusion duration modifies the concentration–time profile by lowering peak concentrations, increasing trough concentrations, and prolonging time above the MIC. While these changes may improve pharmacodynamic target attainment, they may also increase the likelihood of sustained concentrations near toxicity thresholds, particularly at higher doses or in patients with impaired clearance. Despite increasing clinical interest in CI strategies, a quantitative framework that simultaneously evaluates both efficacy and safety remains limited (Taubert et al., 2017; Santimaleeworagun et al., 2021; Barrasa et al., 2019). Most previous studies have focused primarily on pharmacodynamic target attainment without explicitly accounting for toxicity risk. Consequently, the optimal infusion strategy for linezolid in critically ill patients remains uncertain.

To address this gap, we developed a PopPK model and conducted large-scale Monte Carlo simulations to systematically evaluate how infusion duration influences both efficacy and safety targets. Importantly, by introducing a composite efficacy–safety target, this study moves beyond the conventional binary comparison of intermittent versus continuous infusion. Instead, we propose the concept of a dose-dependent optimal infusion duration, recognizing that the most appropriate infusion strategy may vary with the administered dose. By integrating antibacterial efficacy with the risk of drug accumulation, this framework enables a more comprehensive assessment of infusion strategies and facilitates the identification of regimens that balance bacterial eradication with toxicity risk. These findings provide a novel basis for optimizing and individualizing linezolid dosing in critically ill patients.

## 2 Materials and methods

### 2.1 Study population and data collection

This study was a retrospective analysis of prospectively collected clinical and pharmacokinetic data from 20 critically ill adult patients (aged ≥18 years) admitted to the Emergency Intensive Care Unit (EICU) of the First Affiliated Hospital of Bengbu Medical University between January 2017 and January 2021. Pharmacokinetic sampling was performed according to a predefined therapeutic drug monitoring (TDM) protocol implemented in the ICU. Inclusion was limited to adult patients with confirmed or suspected Gram-positive infections, and patients receiving renal replacement therapy were excluded to minimize confounding effects on intrinsic drug clearance. The study protocol was approved by the Ethics Committee of the First Affiliated Hospital of Bengbu Medical University (Approval No. 2017KY008), and informed consent was obtained from all patients or their legal surrogates.

During the study period, a total of 42 ICU patients received linezolid therapy. Among them, 3 patients were excluded because their age did not meet the inclusion criteria, 7 patients were excluded due to oral administration, 2 patients were excluded because their clinical condition prevented continuation of linezolid treatment for more than 12 h, 4 patients were excluded due to concomitant use of medications that might affect the pharmacokinetics of linezolid, and 6 patients were excluded because their families requested withdrawal from the study or declined to provide informed consent. Ultimately, 20 patients were included in the final analysis. All included patients received intravenous linezolid at a dose of 600 mg every 12 h (q12 h) and were stratified according to infusion duration: 10 patients received a 1-h infusion (Standard Infusion group, SI) and 10 patients received a 3-h infusion (PII group). The detailed participant screening and allocation process is shown in Supplementary Figure S1.

Venous blood samples were collected immediately prior to the first administration of linezolid (0 h) and at 0.5, 1, 2, 3, 4, 6, 8, and 12 h after the initiation of intravenous infusion. The 0-h time point represented the pre-dose baseline prior to drug exposure. Plasma samples were separated by centrifugation at 3,000 rpm for 5 min and stored at −80 °C until analysis. Plasma linezolid concentrations were quantified using a validated high-performance liquid chromatography (HPLC) method as previously described (Zhao et al., 2021). Prior to pharmacokinetic analysis, all available concentration data were systematically screened for completeness, plausibility, and analytical reliability. Data quality assessment was performed according to predefined criteria, including consistency with the scheduled sampling scheme and assay performance. Potential outliers were evaluated through graphical inspection and diagnostic analyses, and no implausible observations requiring exclusion were identified. In addition, all covariate data were comprehensively reviewed, and no missing values relevant to the final model were detected; therefore, no data imputation procedures were applied. Consequently, plasma concentration data corresponding to the predefined sampling schedule were retained in the final dataset for population pharmacokinetic modeling. The potential influence of five commonly reported covariates—sex, age, WT, albumin, and CLcr—on pharmacokinetic parameters was evaluated. The selection of these covariates was based on prior pharmacokinetic investigations (Bandín-Vilar et al., 2022; Hui et al., 2022; Qin et al., 2022; Liu et al., 2023; Liu T. et al., 2024). CLcr was estimated using the Cockcroft-Gault equation.

### 2.2 Population pharmacokinetic modeling

PopPK analysis was performed using NONMEM(Bauer, 2019a; Bauer, 2019b) (version 7.5, ICON Development Solutions, USA) assisted by the Perl-speaks-NONMEM (PsN) toolkit (Keizer et al., 2013). The First-Order Conditional Estimation with Interaction (FOCE-I) method was employed for all model development, parameter estimation, and evaluation (Bauer, 2019b).

#### 2.2.1 Base model

Based on the known pharmacokinetic properties of linezolid, as reported in previous pharmacokinetic studies (Matsumoto et al., 2010; Sasaki et al., 2011; Crass et al., 2019; Wang et al., 2021; Bandín-Vilar et al., 2022; Qin et al., 2022; Heidari and Khalili, 2023), both one- and two-compartment models with first-order absorption and elimination were evaluated to determine the optimal structural base model. Inter-individual variability (IIV) in pharmacokinetic parameters was described using an exponential error model (Equation 1):

$$P_{i}=P_{\text{pop}}\times \exp\left(\eta_{i}\right)$$ (1)

where *P*_*i*_ is the parameter value (e.g., CL or V) for the *i*th individual, *P*_pop_ is the typical population value, and $\eta_{i}$ is a random variable normally distributed with a mean of 0 and variance $\omega^{2}$ , representing the deviation of the *i*th individual from the population mean. The magnitude of IIV was expressed as the coefficient of variation (CV%), calculated as CV% = $\sqrt{\omega^{2}}$ × 100%.

During base model development, residual variability was evaluated using additive, exponential, and combined error models. The following exponential error model (Equation 2) was selected as the final model, as it provided better goodness-of-fit and improved model stability:

$$C_{obs,ij}=C_{pred,ij}\times \exp\left(\varepsilon_{ij}\right)$$ (2)

where *C*_*obs,ij*_ and *C*_*pred,ij*_ represent the observed and predicted concentrations for the *i*th individual at the *j*th time point, respectively. $\varepsilon_{ij}$ is a random variable normally distributed with a mean of 0 and variance $\sigma^{2}$ .

#### 2.2.2 Covariate analysis

Prior to covariate selection, pairwise relationships among candidate covariates (sex, age, WT, albumin, and CLcr) were examined using a covariate screening plot. Diagonal panels (Supplementary Figure S2) display variable distributions (histograms for continuous variables and bar plots for categorical variables), lower panels show scatterplots or boxplots with LOESS smoothing colored by sex, and upper panels show Pearson correlation coefficients for continuous variables. The analysis indicated weak correlations among covariates, with a maximum correlation coefficient of 0.31 and a minimum of −0.27, suggesting no evidence of multicollinearity. These results supported the independent evaluation of these covariates in subsequent modeling steps. To identify sources of variability in CL and V, the following covariates were evaluated: WT, age, sex, ALB, and CLcr. Covariate selection was performed using Stepwise Covariate Modeling (SCM).

1. Graphical Exploration: Scatter plots of Empirical Bayes Estimates (ETAs) versus covariates were visually inspected for trends.
2. Forward Addition: Covariates were added sequentially to the base model. Continuous covariates (e.g., WT, CLcr) were modeled using a centered power function (Equation 3):

$$P_{i}=P_{pop}\times {\left(\frac{Cov_{i}}{C{ov}_{median}}\right)}^{\theta }$$ (3)

where Cov_*i*_ represents the value of the covariate for the *i*th individual, *Cov*_*median*_ is the median of this covariate, and *θ* is the influence factor of the covariate. A covariate was considered significant and retained if its inclusion reduced the Objective Function Value (OFV) by > 3.84 (P < 0.05).

3. Backward Elimination: Covariates identified in the forward step were removed one by one from the full model. A stricter criterion of an OFV increase >6.63 (P < 0.01) was required to retain a covariate in the final model.

### 2.3 Model validation

The final model’s stability and predictive performance were assessed using a multi-faceted approach.

1. Goodness-of-Fit (GOF) Plots: Visual diagnostics included: (a) Observed (OBS) vs. Population Predicted (PRED) concentrations; (b) OBS vs. Individual Predicted (IPRED) concentrations; (c) Conditional Weighted Residuals (CWRES) vs. PRED; and (d) CWRES vs. Time. Ideally, the scattered points of (a) and (b) should be evenly distributed on both sides of the *y* = *x* diagonal. The scattered points of (c) and (d) should be evenly distributed on both sides of the *y* = 0 horizontal line, with no obvious trend.
2. Bootstrap Analysis: This is the gold standard method for evaluating the uncertainty and stability of model parameters (Bauer, 2019b). With replacement, repeated sampling was conducted from the original dataset of 20 patients to generate 1,000 new datasets of the same size as the original. The final PPK model and parameter structure were refitted to these 1,000 Bootstrap datasets. Then, the median and 95% confidence intervals (CI) of all model parameter estimates were calculated. If the parameter estimates of the original model all fall within the 95% confidence intervals calculated by the Bootstrap method and the interval does not contain 0 (for effect coefficients) or is within a reasonable range, it indicates that the model is stable and robust.
3. Visual Predictive Check (VPC): To comprehensively evaluate the final population pharmacokinetic model’s descriptive ability for the original data and the fidelity of its simulation predictions, virtual data consistent with the structure of the original dataset is generated using the final model parameters. By calculating the 95% confidence intervals (i.e., prediction intervals) of the fifth, 50th (median), and 95th percentiles of the simulated data at each time point and comparing them with the corresponding percentile lines of the measured data. If the observed percentile curves fall within the corresponding simulation-based confidence intervals without systematic bias, the model is considered to adequately describe both the central tendency and variability of the data. This step not only validates the goodness of fit but also provides a solid methodological basis for subsequent Monte Carlo simulations based on this model.

### 2.4 Monte Carlo simulation and dosing design

Monte Carlo simulations were conducted to evaluate the PTA for various dosing strategies.

1. Virtual Population: To evaluate the PTA, 1,000 virtual critically ill patients were generated using demographic variances (Mean WT = 61.5 kg; Mean CLcr = 106.5 mL/min) derived from the study population.
2. Dosing Matrix: To systematically evaluate the impact of dose and infusion duration, a dosing matrix was constructed. Maintaining a fixed dosing frequency of every 12 h (q12 h), the simulation evaluated a combinatorial design of 7 dose levels (300–900 mg, in 100 mg increments) and 13 infusion durations (0.5, 1, 2, … , 12 h). This design encompassed the full spectrum of administration strategies, ranging from standard short infusions to CI (12 h).
3. Virtual pathogens: The simulation was also conducted for virtual pathogens with MIC values of 0.125, 0.25, 0.5, 1, 2, 3, 4 mg/L. MIC values up to 4 mg/L were evaluated in the simulations, as this range reflects the clinically relevant susceptibility spectrum for linezolid and corresponds to the EUCAST clinical breakpoint (European Committee on Antimicrobial Susceptibility Testing of the European Society of Clinical and Infectious, 2001). Pathogens with MIC values exceeding this threshold are generally considered less suitable for linezolid therapy; therefore, higher MIC values were not routinely simulated.

### 2.5 PK/PD analysis and target definition

To ensure the representativeness of the data, the calculations of all key PK/PD indicators (such as steady-state trough concentration C_min,ss_ and the area under the 24-h steady-state plasma concentration-time curve AUC_24,ss_) were all derived from the simulation data after reaching steady state on the seventh day (144–168 h).

#### 2.5.1 Calculation of PK/PD indices

Steady-state pharmacokinetic profiles were simulated for each virtual patient. Key indices were calculated via numerical integration.

1. AUC_24,ss_: Area under the concentration-time curve over 24 h at steady state, representing total drug exposure.
2. C_min,ss_: Minimum plasma concentration at the end of the dosing interval at steady state, used to assess accumulation.

#### 2.5.2 Definition of the composite efficacy-safety target

To address the narrow therapeutic window of linezolid, a strict composite efficacy–safety target was defined to guide dosing optimization.

1. Efficacy target: Based on linezolid’s pharmacodynamic characteristics, efficacy was defined as the simultaneous attainment of AUC_24,ss_/MIC ≥100 and %T > MIC ≥85%, thresholds associated with bacterial eradication and favorable clinical outcomes (Lau et al., 2023; Blackman et al., 2021).
2. Safety target: To minimize mitochondrial toxicity associated with sustained high trough concentrations, such as myelosuppression and lactic acidosis, a safety threshold of C_min,ss_ < 8 mg/L was applied based on previously reported exposure–toxicity relationships (Matsumoto et al., 2014; Boak et al., 2014; Bandín-Vilar et al., 2022).
3. PTA: PTA was defined as the proportion of simulated patients simultaneously achieving both efficacy and safety targets for a given dosing regimen and MIC.
4. Failure analysis: Treatment failure was categorized as either under-exposure (efficacy target not achieved) or over-exposure (safety threshold exceeded, C_min,ss_ ≥ 8 mg/L).

## 3 Results

### 3.1 Patient demographics

Twenty critically ill patients were enrolled for pharmacokinetic analysis according to the predefined sampling schedule. The cohort was gender-balanced (55% male) and exhibited substantial physiological heterogeneity, particularly regarding renal function (Table 1). CLcr ranged from 71 to 128 mL/min, capturing a broad spectrum of renal physiology from mild impairment to the threshold of ARC. This pathophysiological variability ensured that the resulting PopPK model incorporated the diverse clinical scenarios characteristic of the ICU population.

**TABLE 1:** Patient characteristics used to evaluate population model.

Characteristics	Number or mean ± SD	Median (range)
1-h Infusion		
No. Of patients (male/Female)	10 (7/3)	—
No. Of samples	90	—
Age (years)	60.8 ± 6.1	62 (51–68)
Weight (kg)	65.2 ± 11.7	62 (55–96)
Albumin (g/L)	44.1 ± 6.0	44.85 (33.9–50.6)
Creatinine clearance (mL/min)	107.2 ± 14	110 (71–124)
3-h Infusion		
No. Of patients (male/Female)	10 (4/6)	—
No. Of samples	90	—
Age (years)	55.7 ± 8.2	55 (40–67)
Weight (kg)	58.9 ± 4.7	59 (52–65)
Albumin (g/L)	43.0 ± 6.9	44.1 (26.9–52.4)
Creatinine clearance (mL/min)	99.1 ± 17.6	97.5 (72–128)

### 3.2 Population pharmacokinetic modeling

#### 3.2.1 Base model selection

Structural model evaluation compared one- and two-compartment specifications. Although the two-compartment model significantly reduced the OFV (338.78 vs. 380.62; ΔOFV = −41.84), it compromised parameter stability. This was evidenced by a substantial increase in the Relative Standard Error (RSE) of the primary fixed effect (from 8.7% to 58.5%) and an excessive Condition Number, suggesting potential over-parameterization and limited parameter identifiability. Given the instability of the two-compartment parameters, the lower OFV was considered insufficient to justify the additional structural complexity. We also acknowledge that ICU patients may exhibit altered distribution kinetics. However, the primary objective of this study was to support dosing simulations focused on steady-state exposure metrics (AUC and C_min_). In this context, the predictive performance of the one-compartment model was comparable to that of the two-compartment model. Considering the instability of the additional parameters in the two-compartment model, the more parsimonious structure was therefore selected to avoid potential overfitting. Therefore, following the principle of parsimony and prioritizing model robustness, the basic one-compartment model with zero-order input and first-order elimination was selected as the structural base model. While this model estimated IIV for V and CL with reasonable precision, GOF diagnostics (Supplementary Figure S3) revealed systematic bias in Conditional Weighted Residuals (CWRES), characterized by over-prediction at low concentrations and under-prediction at high concentrations, indicating the need for subsequent covariate analysis to further improve model performance.

#### 3.2.2 Covariate model

Covariate analysis evaluated WT, age, sex, ALB, and CLcr. Initial graphical exploration (Supplementary Figure S4) suggested correlations between WT and V, and between CLcr and CL. SCM confirmed these relationships: Forward Step (P < 0.05): WT and CLcr were identified as significant covariates for V and CL, respectively. Backward Step (P < 0.01): Both covariates were retained. The main steps from the base model to the final model are summarized in Supplementary Table S1. The final covariate model was described using power functions: V(L) = 30.7×(WT/61.5)^2.16^, CL (L/h) = 7.37×(CLcr/106.5)^1.35^. As shown in Supplementary Figure S4, the inclusion of these covariates significantly reduced the trend in IIV, confirming their physiological plausibility. Furthermore, as detailed in Table 2, the RSEs for the typical values of V and CL decreased from 8.7% to 7.1%–6% and 5.2% respectively, compared to the base model, indicating substantially improved estimation precision.

**TABLE 2:** Population pharmacokinetic parameter estimates and bootstrap evaluation.

Parameters	Base model		Final model		
	Parameter estimates (%CV)	Shrinkage (%)	Parameter estimates (%CV)	Shrinkage (%)	Bootstrap Median (2.5%–97.5%)
V(L)	30.9 (8.7)	—	30.7 (6)	—	30.67 (26.96–34.98)
WT_V	—	—	2.16 (21.5)	—	2.19 (0.51–2.99)
CL (L/h)	6.96 (7.1)	—	7.37 (5.2)	—	7.36 (6.53–8.25)
CLcr_CL	—	—	1.35 (22.9)	—	1.35 (0.72–1.93)
Between-subject variability (%CV)					
ωV	37.3 (17.2)	2	24.1 (19.7)	6.6	22.83 (5.94–30.52)
ωCL	30.4 (17.1)	1.4	20.8 (18.2)	4.8	19.46 (14.3–23.69)
Residual unexplained variability					
ε	21.63 (13.4)	11.1	21.7 (13.4)	10.1	21.79 (19.71–23.77)

#### 3.2.3 Model validation

**FIGURE 1:**
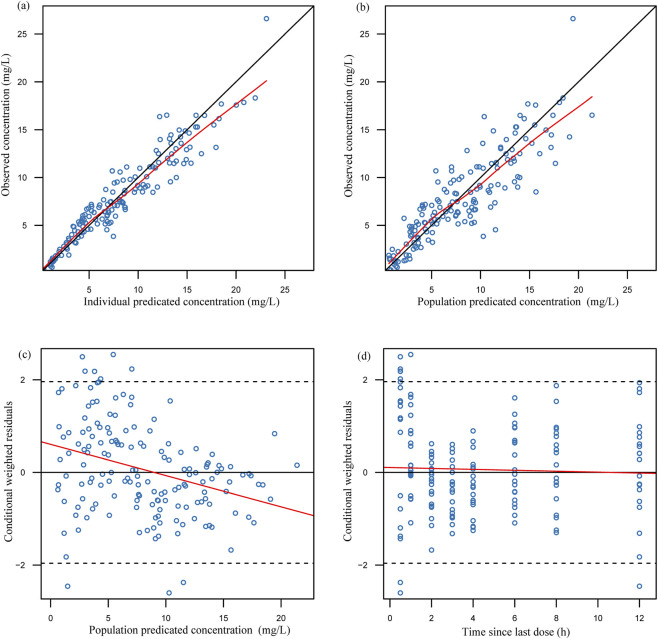
Goodness-of fit plots obtained from the final linezolid population pharmacokinetic model. **(a)** Observation concentration vs. individual predicated concentration, **(b)** Observation concentration vs. population predicated concentration, **(c)** Conditional weighted residuals vs. the population predictions, **(d)** Conditional weighted residuals vs. the time after dosing. The red curves represent the locally weighted scatterplot smoothing lines.

**FIGURE 2:**
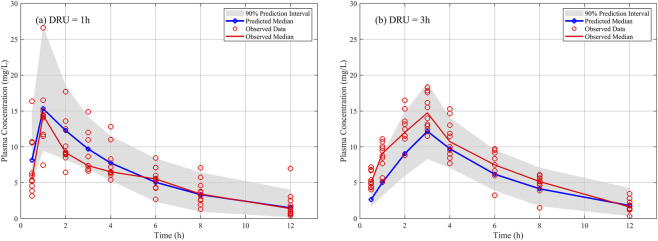
Visual predictive check of the final model. Shaded areas represent nonparametric 90% confidence intervals of the predicted concentrations. The blue lines represent the median of the predicted data. Red circles represent observed data. Red lines represent the median of the observed data. **(a)** DRU = 1h. **(b)** DRU = 3h.

GOF diagnostics (Figure 1) indicated strong concordance between observed concentrations and both population (PRED) and individual (IPRED) predictions. Data points were symmetrically distributed around the line of identity, showing no evidence of systematic bias. CWRES were randomly distributed around zero, predominantly falling within the ±2 range. Assessing model stability, the 1,000-run Bootstrap analysis achieved full convergence; shrinkage for IIV and residual variability remained below 30%, and all final parameter estimates were contained within the Bootstrap 95% confidence intervals (deviation <7%). Furthermore, VPC (Figure 2) demonstrated that the model-simulated median and 90% prediction intervals accurately captured the central tendency and dispersion of the observed data. Collectively, these validation metrics substantiate the model’s robustness and predictive accuracy for subsequent simulations.

### 3.3 Impact of infusion duration on steady-state pharmacokinetics

**FIGURE 3:**
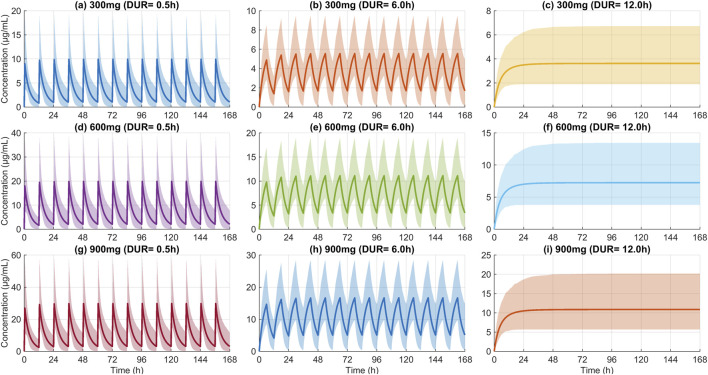
The Monte Carlo simulation of the serum concentration-time curve after intravenous administration of linezolid: **(a)** 300 mg (administered by 0.5-h infusion) q 12 h, **(b)** 300 mg (administered by 6-h infusion) q 12 h, **(c)** 300 mg (administered by 12-h infusion) q 12 h, **(d)** 600 mg (administered by 0.5-h infusion) q 12 h, **(e)** 600 mg (administered by 6-h infusion) q 12 h, **(f)** 600 mg (administered by 12-h infusion) q 12 h, **(g)** 900 mg (administered by 0.5-h infusion) q 12 h, **(h)** 900 mg (administered by 6-h infusion) q 12 h, **(i)** 900 mg (administered by 12-h infusion) q 12 h. The solid lines represent the median, and the shaded areas indicate the 95% confidence interval of the simulated concentration.

Monte Carlo simulations quantified the modulation of steady-state (144–168 h) pharmacokinetics by infusion duration. As illustrated in Figure 3, extending the infusion duration significantly attenuated peak-to-trough fluctuations. Short infusions (0.5–1 h) resulted in pronounced concentration excursions characterized by high C_max_ and low C_min_. As infusion duration increased, the amplitude of fluctuation narrowed, eventually converging to an invariant steady-state concentration at 12 h. This study firstly provides the quantitative characterization of the nonlinear kinetics of concentration extremes relative to infusion duration. Simulations demonstrated that C_min,ss_ exhibited a nonlinear increase, while C_max,ss_ showed a nonlinear decay with increasing duration (Supplementary Figure S5). The fitted relationship is described by (Equation 4):

$$\begin{array}{l} C_{\min,\text{ss}}=\frac{\text{Dose}}{300}\times 0.67\times e^{0.1308\times \text{DUR}}\\ C_{\max,\text{ss}}=\frac{\text{Dose}}{300}\times 9.158\times e^{-0.0878\times \text{DUR}} \end{array}$$ (4)

These findings demonstrate that while C_min,ss_ and C_max,ss_ are linearly related to total dose, they exhibit a nonlinear dependence on infusion duration. Conversely, steady-state daily exposure (AUC_24,ss_) showed complete dose-dependency (AUC_24,ss_ = 0.2687 × Dose) but remained independent of infusion duration (Supplementary Figure S6). This confirms pharmacokinetically that extended infusion represents an “iso-exposure, variable-distribution” strategy: its primary therapeutic value lies in optimizing the temporal distribution of the drug within the dosing interval without altering the total systemic burden.

### 3.4 PTA under composite targets

**FIGURE 4:**
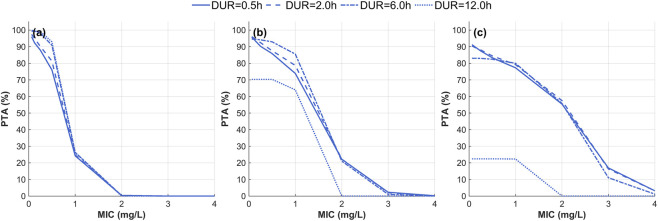
PTA of various linezolid dosing regimens with MIC (mg/L). **(a)** 300 mg q 12 h. **(b)** 600 mg q 12 h. **(c)** 900 mg q 12 h.

**FIGURE 5:**
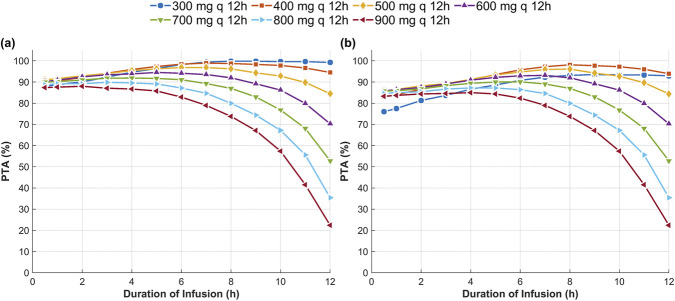
PTA of various linezolid dosing regimens with duration of infusion for low MIC (mg/L). **(a)** MIC=0.25 mg/L. **(b)** MIC=0.50 mg/L.

**FIGURE 6:**
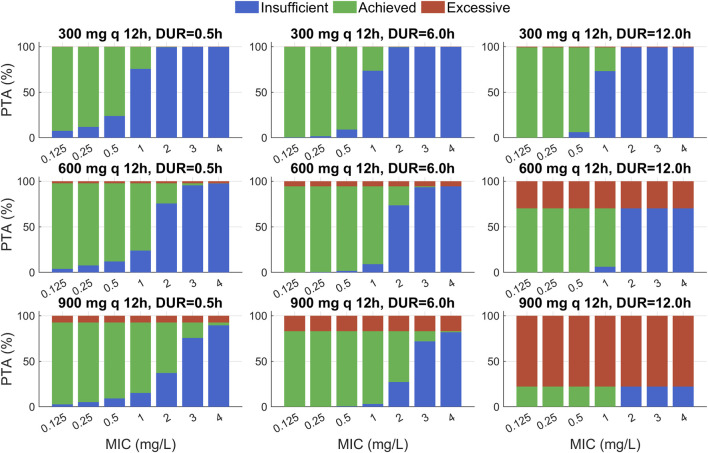
Stacked bar graph of the PTA of various linezolid dosing regimens with MIC.

**FIGURE 7:**
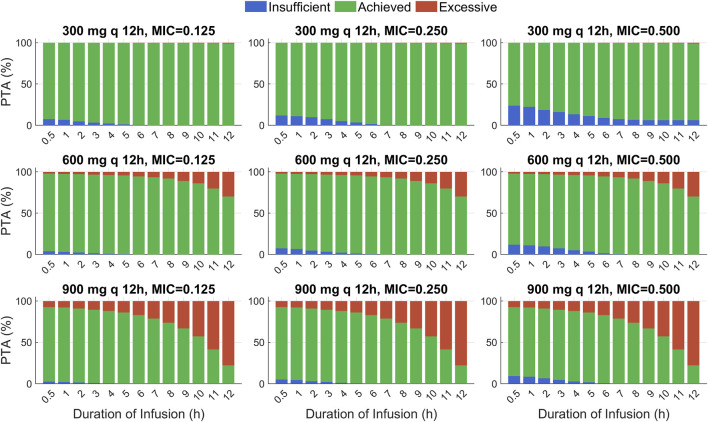
Stacked bar graph of the PTA of various linezolid dosing regimens with duration of infusion for low MIC (mg/L).

Figures 4–7 and Supplementary Figure S7, S8 present the PTA analysis under the dual constraints of efficacy and safety. The results reveal that the relationship between infusion duration and PTA is nonlinear and strictly dose-dependent. Low-Dose Regimens (e.g., 300 mg q12h, Figure 4a): PTA increased monotonically with infusion duration. CI was clearly the optimal strategy. This is because, at low doses, the primary limiting factor is insufficient efficacy; thus, extending infusion maximizes % T > MIC. Moderate-to-High Dose Regimens (≥600 mg q12h, Figures 4b,c): The trend reversed. CI resulted in significantly lower PTA compared to intermittent infusion, a disadvantage that worsened with increasing dose. Figure 5 and Supplementary Figure S7 further illuminate this relationship: PTA exhibited a non-monotonic relationship as infusion duration increased. This indicates the existence of an “Optimal Duration Range” rather than a “longer is better” rule. Specifically, as infusion duration increased from 0.5 h to 2–5 h, PTA rose due to improved efficacy. However, extending beyond 6 h (especially to CI) caused a significant decline in PTA.

The mechanism underlying this phenomenon is the trade-off between “efficacy benefit” and “toxicity risk”, as revealed by the stacked bar charts (Figures 6, 7; Supplementary Figure S8). While extending infusion reduced the proportion of efficacy failure (blue area), it caused a sharp increase in the risk of over-exposure (C_min,ss_ ≥ 8 mg/L, red area), which negated the efficacy gains. This conflict is most acute for high-resistance pathogens (MIC ≥2 mg/L, Table 3). In this scenario, standard dosing is ineffective, yet high-dose CI results in a PTA near 0% due to the simultaneous presence of “insufficient efficacy” and “excessive toxicity”. Comprehensive simulations suggest that for refractory infections (MIC ≥2 mg/L), only high-dose regimens combined with a specific prolonged intermittent infusion duration (e.g., 2–5 h) can synergistically maximize efficacy while keeping toxicity risks within an acceptable range. These warn against the indiscriminate application of CI for high-dose linezolid, advocating instead for a precise, dose-dependent optimization of infusion duration.

**TABLE 3:** Probability of target attainment of various linezolid dosing regimens at several PK/PD targets.

Dosing regimen	MIC = 0.125 mg/L	MIC = 0.25 mg/L	MIC = 0.5 mg/L	MIC = 1 mg/L	MIC = 2 mg/L	MIC = 4 mg/L
300 mg q 12 h						
DUR = 0.5 h	92.3 (↓7.6; ↑0.1)	87.9 (↓12; ↑0.1)	76 (↓23.9; ↑0.1)	24.2 (↓75.7; ↑0.1)	0.4 (↓99.5; ↑0.1)	0 (↓99.9; ↑0.1)
DUR = 3 h	96.6 (↓3.3; ↑0.1)	92.3 (↓7.6; ↑0.1)	83.7 (↓16.2; ↑0.1)	25.2 (↓74.7; ↑0.1)	0.4 (↓99.5; ↑0.1)	0 (↓99.9; ↑0.1)
DUR = 6 h	99.4 (↓0.4; ↑0.2)	98.2 (↓1.6; ↑0.2)	90.8 (↓9; ↑0.2)	26.3 (↓73.5; ↑0.2)	0.3 (↓99.5; ↑0.2)	0 (↓99.8; ↑0.2)
DUR = 9 h	99.8 (↓0; ↑0.2)	99.8 (↓0; ↑0.2)	93.5 (↓6.3; ↑0.2)	26.7 (↓73.1; ↑0.2)	0.3 (↓99.5; ↑0.2)	0 (↓99.8; ↑0.2)
DUR = 12 h	99.2 (↓0; ↑0.8)	99.2 (↓0; ↑0.8)	92.9 (↓6.3; ↑0.8)	26.1 (↓73.1; ↑0.8)	0 (↓99.2; ↑0.8)	0 (↓99.2; ↑0.8)
500 mg q 12 h						
DUR = 0.5 h	95.0 (↓4.6; ↑0.4)	91.0 (↓8.6; ↑0.4)	85.8 (↓13.8; ↑0.4)	67.8 (↓31.8; ↑0.4)	11.6 (↓88; ↑0.4)	0 (↓99.6; ↑0.4)
DUR = 3 h	96.3 (↓2.1; ↑1.6)	94 (↓4.4; ↑1.6)	89.2 (↓9.2; ↑1.6)	74.1 (↓24.3; ↑1.6)	11.2 (↓87.2; ↑1.6)	0 (↓98.4; ↑1.6)
DUR = 6 h	97.2 (↓0.1; ↑2.7)	96.8 (↓0.5; ↑2.7)	94.7 (↓2.6; ↑2.7)	78.1 (↓19.2; ↑2.7)	10.4 (↓86.9; ↑2.7)	0 (↓97.3; ↑2.7)
DUR = 9 h	94.3 (↓0; ↑5.7)	94.3 (↓0; ↑5.7)	94.2 (↓0.1; ↑5.7)	77.3 (↓17; ↑5.7)	7.6 (↓86.7; ↑5.7)	0 (↓94.3↑5.7)
DUR = 12 h	84.5 (↓0.1; ↑15.5)	84.5 (↓0; ↑15.5)	84.4 (↓0.1; ↑15.5)	67.5 (↓17; ↑15.5)	0 (↓84.5; ↑15.5)	0 (↓84.5; ↑15.5)
700 mg q 12 h						
DUR = 0.5 h	93.3 (↓3.2; ↑3.5)	90 (↓6.5; ↑3.5)	85.8 (↓10.7; ↑3.5)	76.9 (↓19.6; ↑3.5)	33 (↓63.5; ↑3.5)	0.2 (↓96.3; ↑3.5)
DUR = 3 h	93.4 (↓1.3; ↑5.3)	91.8 (↓2.9; ↑5.3)	88.3 (↓6.4; ↑5.3)	82.2 (↓12.5; ↑5.3)	34.6 (↓60.1; ↑5.3)	0.1 (↓94.6; ↑5.3)
DUR = 6 h	91.3 (↓0; ↑8.7)	91.1 (↓0.2; ↑8.7)	90.1 (↓1.2; ↑8.7)	86.2 (↓5.1; ↑8.7)	33 (↓58.3; ↑8.7)	0.1 (↓91.2; ↑8.7)
DUR = 9 h	83 (↓0; ↑17)	83 (↓0; ↑17)	83 (↓0; ↑17)	81.1 (↓1.9; ↑17)	24.9 (↓58.1; ↑17)	0 (↓83; ↑17)
DUR = 12 h	52.8 (↓0; ↑47.2)	52.8 (↓0; ↑47.2)	52.8 (↓0; ↑47.2)	50.9 (↓1.9; ↑47.2)	0 (↓52.8; ↑47.2)	0 (↓52.8; ↑47.2)
900 mg q 12 h						
DUR = 0.5 h	89.9 (↓2.7; ↑7.4)	87.4 (↓5.2; ↑7.4)	83.3 (↓9.3; ↑7.4)	77.3 (↓15.3; ↑7.4)	55.4 (↓37.2; ↑7.4)	3.2 (↓89.4; ↑7.4)
DUR = 3 h	88.5 (↓1; ↑10.5)	87.1 (↓2.4; ↑10.5)	84.6 (↓4.9; ↑10.5)	79.5 (↓10; ↑10.5)	58.1 (↓31.4; ↑10.5)	2.9 (↓86.6; ↑10.5)
DUR = 6 h	83 (↓0; ↑17)	82.9 (↓0.1; ↑17)	82.4 (↓0.6; ↑17)	80 (↓3; ↑17)	55.8 (↓27.2; ↑17)	1.2 (↓81.8; ↑17)
DUR = 9 h	67.1 (↓0; ↑32.9)	67.1 (↓0; ↑32.9)	67.1 (↓0; ↑32.9)	67 (↓0.1; ↑32.9)	41.7 (↓25.4; ↑32.9)	0 (↓67.1; ↑32.9)
DUR = 12 h	22.4 (↓0; ↑77.6)	22.4 (↓0; ↑77.6)	22.4 (↓0; ↑77.6)	22.3 (↓0.1; ↑77.6)	0 (↓22.4; ↑77.6)	0 (↓22.4; ↑77.6)

DUR–duration of infusion; data are expressed as percentage of patient in the therapeutic range (that achieve the PK/PD, target but remain below the toxicity threshold), ↓—percentage of patient below the range (not achieving the PK/PD, target); ↑—percentage of patient above the range (exceeding the toxicity threshold).

## 4 Discussion

This study systematically investigated the complex impact of infusion duration on a composite efficacy–safety target using a rigorously validated PopPK model combined with large-scale Monte Carlo simulations. Our findings indicate that CI is not universally optimal, and that the most appropriate infusion strategy depends on the specific dose–MIC context. In other words, we propose a precision dosing framework tailored to different dose–MIC scenarios. By evaluating the trade-off between PTA and toxicity risk across varying infusion durations, this study provides a practical framework for individualized linezolid dosing in critically ill patients.

### 4.1 Model validity

The one-compartment PopPK model developed in this study provides a pharmacokinetic description that is broadly consistent with previously published models of linezolid in critically ill populations, supporting its use as a framework for simulation-based analyses. Although the present study included a relatively small cohort, the structural model and key covariate relationships were generally aligned with existing pharmacokinetic evidence. In particular, the linear one-compartment structure has been widely reported in population pharmacokinetic analyses of linezolid in ICU patients and other clinical populations (Sasaki et al., 2011; Boak et al., 2014; Wang et al., 2021). Similarly, the identification of CLcr as a significant predictor of systemic clearance is biologically plausible and has been consistently reported in previous studies (Matsumoto et al., 2014; Wang et al., 2021). WT as a determinant of the volume of distribution has also been described in earlier pharmacokinetic analyses (Sasaki et al., 2011; Abe et al., 2009; Tietjen et al., 2022). Importantly, the typical pharmacokinetic parameter estimates obtained in our study (CL = 7.06 L/h; V = 31.3 L) fall within the ranges reported in comparable cohorts of critically ill patients (Soraluce et al., 2020; Wang et al., 2021), further supporting the physiological plausibility and external consistency of the model.

Beyond parameter estimation, the model enabled exploration of how infusion duration influences the resulting concentration–time profiles. As illustrated in Figure 3, extending the infusion duration progressively attenuates peak-to-trough fluctuations in plasma concentrations. When the infusion duration approaches continuous administration, the concentration profile gradually converges toward a near-steady-state pattern. This kinetic behavior is consistent with recent experimental and simulation data reported by Zavřelová et al. (2025) (Zavřelová et al., 2025). Importantly, our simulations (Supplementary Figure S5) confirmed that the steady-state exposure (AUC_24,ss_) remained primarily determined by the administered dose and systemic clearance and therefore increased proportionally with dose, consistent with previous observations reported by Pea et al. (Pea et al., 2012; Pea et al., 2017). In contrast, the concentration extremes at steady state (C_max,ss_ and C_min,ss_) demonstrated a pronounced nonlinear sensitivity to infusion duration (Supplementary Figure S6). As infusion time increased, peak concentrations decreased while trough concentrations increased, resulting in progressive compression of peak-to-trough fluctuations.

A distinctive aspect of the present study is the derivation of predictive equations that quantitatively describe this nonlinear compression phenomenon. By explicitly linking infusion duration with steady-state concentration extremes, the proposed framework provides a practical computational tool for anticipating how changes in infusion strategy may reshape the concentration profile. This extends previous research that has predominantly focused on AUC-based exposure metrics while providing limited characterization of how dosing strategies influence concentration variability across the dosing interval. It should be noted that, although pharmacokinetic metrics such as C_max_, C_min,_ and AUC can be derived analytically based on model parameters, these deterministic relationships do not account for interindividual variability and therefore cannot be used to estimate the probability of target attainment. The analytical equations provide a convenient and rapid means to estimate these metrics, facilitating identification of optimal infusion strategies at the individual level. In contrast, Monte Carlo simulations were employed to incorporate variability and parameter uncertainty, enabling a clinically relevant evaluation of dosing strategies at the population level. Thus, the analytical relationships and simulation-based analysis serve complementary roles: the former supports rapid individual-level estimation, whereas the latter allows assessment of target attainment across a heterogeneous patient population.

### 4.2 Critical selection of pharmacodynamic targets

Establishing a composite target that simultaneously reflects efficacy and safety is fundamental for dosing optimization. For efficacy, we adopted the dual PK/PD standard of AUC_24,ss_/MIC ≥100 and %T > MIC ≥85%, consistent with expert consensus recommendations (Abdul-Aziz et al., 2020; Heidari and Khalili, 2023; Bandín-Vilar et al., 2022). However, the definition of safety thresholds remains controversial. Some studies have proposed an exposure cap of AUC_24,ss_ > 300 mg h/L as a predictor of toxicity (Abdul-Aziz et al., 2020; Zavřelová et al., 2025). Nevertheless, reliance solely on AUC may be theoretically insufficient because it reflects cumulative exposure but does not capture temporal concentration dynamics. Accumulating evidence suggests that linezolid-induced thrombocytopenia is more strongly associated with elevated C_min_ (Matsumoto et al., 2010; Pea et al., 2012). Reported thresholds vary across studies, ranging from approximately <8.2 mg/L (Matsumoto et al., 2014) to stricter ranges of 2–7 mg/L recommended by the European Society of Intensive Care Medicine (Abdul-Aziz et al., 2020). Considering these findings and recent expert consensus on therapeutic drug monitoring, we adopted the conservative threshold of C_min,ss_ < 8 mg/L in this study (Lin et al., 2022). Importantly, this value should be interpreted as a conservative safety reference rather than an absolute toxicity boundary, given that thrombocytopenia risk may vary across patient populations and clinical conditions. Although composite PK/PD targets may be complex for direct clinical monitoring, they provide a comprehensive framework for evaluating the efficacy–safety balance of different dosing strategies. Given the known concentration-dependent toxicity of linezolid, consideration of both efficacy and safety is essential when optimizing dosing. In routine clinical practice, simplified indicators such as C_min_ (e.g., 2–8 mg/L) may remain more practical for therapeutic drug monitoring, while the composite target can be used to inform dosing strategy selection.

### 4.3 PTA analysis and the dose-dependency of infusion duration

The most significant finding of this study is the “context-dependency” of infusion optimization. Simulations clearly demonstrate that the PTA curve evolves from a “monotonic increase” to a “non-monotonic relationship” as dose and MIC increase, effectively debunking the binary view that CI is inherently superior.

1. Linear Benefit at Low Doses: When treating susceptible pathogens with standard doses (≤400 mg q12 h), the primary limitation is insufficient exposure. Here, extending infusion significantly boosts % T > MIC with negligible toxicity risk. Consequently, PTA increases monotonically from short to CI, explaining why previous studies focused on standard doses favored CI strategies (Abou Warda et al., 2022; Hui et al., 2024).
2. Toxicity Reversal at High Doses: Conversely, when escalating doses (≥600 mg q12 h) to combat high-MIC (≥2 mg/L) pathogens, the scenario reverses. The bottleneck shifts from efficacy to safety. The indiscriminate application of CI systematically raises C_min,ss_ above the 8 mg/L red line. The results reveal that at 600 mg q12h, the over-exposure rate surges from 2.2% (0.5 h infusion) to 29.7% (CI), driving a precipitous decline in composite PTA. Even under a relaxed threshold of 10 mg/L, CI retains a significantly higher risk profile (13.3% vs. 0.4%), consistent with findings by Taubert et al. (2017). In addition, Santimaleeworagun et al. (2021) also obtained a similar conclusion in 2021: the probability of side effect risk from CI is higher than that from intermittent infusion.
3. The Optimal Duration Range: High-dose therapy is often required for MIC ≥2 mg/L (Dong et al., 2016; Soraluce et al., 2020; Simon et al., 2020), yet it carries inherent safety risks (Barrasa et al., 2019). To resolve this paradox, we propose an “Optimal Duration Range” (typically 2–5 h). This strategy of PII exploits pharmacokinetic principles: it secures the pharmacodynamic benefits of extended exposure (%T > MIC) while preserving a critical elimination phase at the end of the dosing cycle, allowing C_min,ss_ to recede into the safe range. This aligns with Zheng et al. (2020), suggesting that for intensified regimens, prolonged intermittent infusion offers a superior therapeutic index compared to CI.

### 4.4 Limitations and future perspectives

Although this study provides a PK\PD framework for optimizing linezolid infusion strategies in critically ill patients, several limitations should be acknowledged.

First, the PopPK model was developed from a relatively small cohort (n = 20). Although internal validation using bootstrap analysis indicated satisfactory parameter stability and robustness, the limited sample size may restrict the representation of inter-individual variability and population heterogeneity. Consequently, the generalizability of the model may be limited, and external validation using larger, multicenter cohorts will be necessary to confirm its applicability across broader ICU populations.

Second, the safety evaluation in this study was primarily defined using the C_min,ss_ threshold associated with linezolid-induced myelosuppression. While this approach reflects commonly used clinical monitoring practices, it may not fully capture toxicity risks related to peak exposure (C_max,ss_) or cumulative systemic exposure (e.g., AUC). Future studies incorporating multiple exposure metrics may provide a more comprehensive characterization of exposure–toxicity relationships.

Third, the dosing strategies proposed in this study were derived from Monte Carlo simulations based on the developed PopPK model rather than direct exposure–response or exposure–toxicity analyses within the current cohort. Therefore, these findings should be interpreted as hypothesis-generating rather than definitive clinical recommendations. Prospective studies integrating pharmacokinetic modeling with clinical outcomes—including microbiological response, clinical cure, and adverse events—are warranted to determine whether the proposed dose–duration optimization strategy translates into improved clinical outcomes.

Finally, the simulations assumed a fixed protein binding fraction and did not account for potential variability in protein binding among critically ill patients. Given the high prevalence of hypoalbuminemia in ICU populations, alterations in protein binding may influence free drug exposure and pharmacodynamic target attainment. Future pharmacokinetic models incorporating albumin as a covariate or directly modeling unbound drug concentrations may further refine dosing optimization strategies. In addition, the integration of TDM into model-informed precision dosing frameworks may facilitate dynamic adjustment of infusion duration according to real-time changes in renal function and individual pharmacokinetic variability.

## 5 Conclusion

This study suggests that the optimal infusion strategy for linezolid in critically ill patients is not universal but depends on the interaction between dosing intensity, pathogen susceptibility, and patient pharmacokinetics. CI may improve pharmacodynamic target attainment under standard dosing conditions. However, when higher exposure is required to treat pathogens with elevated MIC values (e.g., MIC ≥2 mg/L), CI may increase the likelihood of excessive drug exposure, particularly in patients with reduced or variable clearance. In these situations, PII (two to five h) may offer a more favorable balance between sustained pharmacodynamic exposure and controlled drug accumulation by allowing a partial elimination phase. These findings highlight the importance of integrating both dose and infusion duration when optimizing linezolid therapy in critically ill patients. Overall, our results support a context-dependent dosing strategy, in which infusion modality is individualized according to pharmacokinetic variability and pathogen susceptibility, ideally guided by TDM to maximize efficacy while minimizing toxicity.

## Data Availability

The data analyzed in this study is subject to the following licenses/restrictions: Data from this study cannot be shared publicly because of the privacy statement included in the consent documents signed by the parents or legal guardians of the patients involved in this research. Requests to access these datasets should be directed to CW, 012014041@bbmu.edu.cn.
