# Supplementary material for: Optimization of linezolid infusion duration in critically ill patients: a population pharmacokinetic analysis of the trade-off between efficacy and toxicity
Source: Front Pharmacol. 2026 Jun 30;17:1766346. doi: 10.3389/fphar.2026.1766346 (PMC13364645; doi:10.3389/fphar.2026.1766346)
Supplement: Supplementary file 1 [file Supplementaryfile1.docx]

Supplementary Material

# Supplementary Figures and Tables

## Supplementary Figures


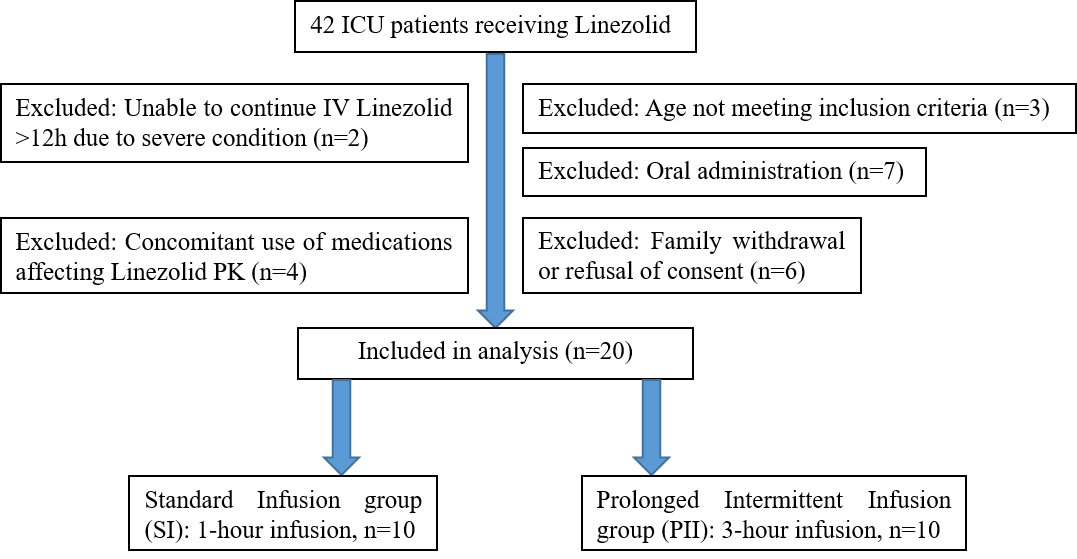


**Supplementary Figure 1. Flow diagram of patient inclusion, exclusion, and allocation to infusion groups.** Abbreviations: SI, standard infusion; PII, prolonged intermittent infusion; ICU, intensive care unit.


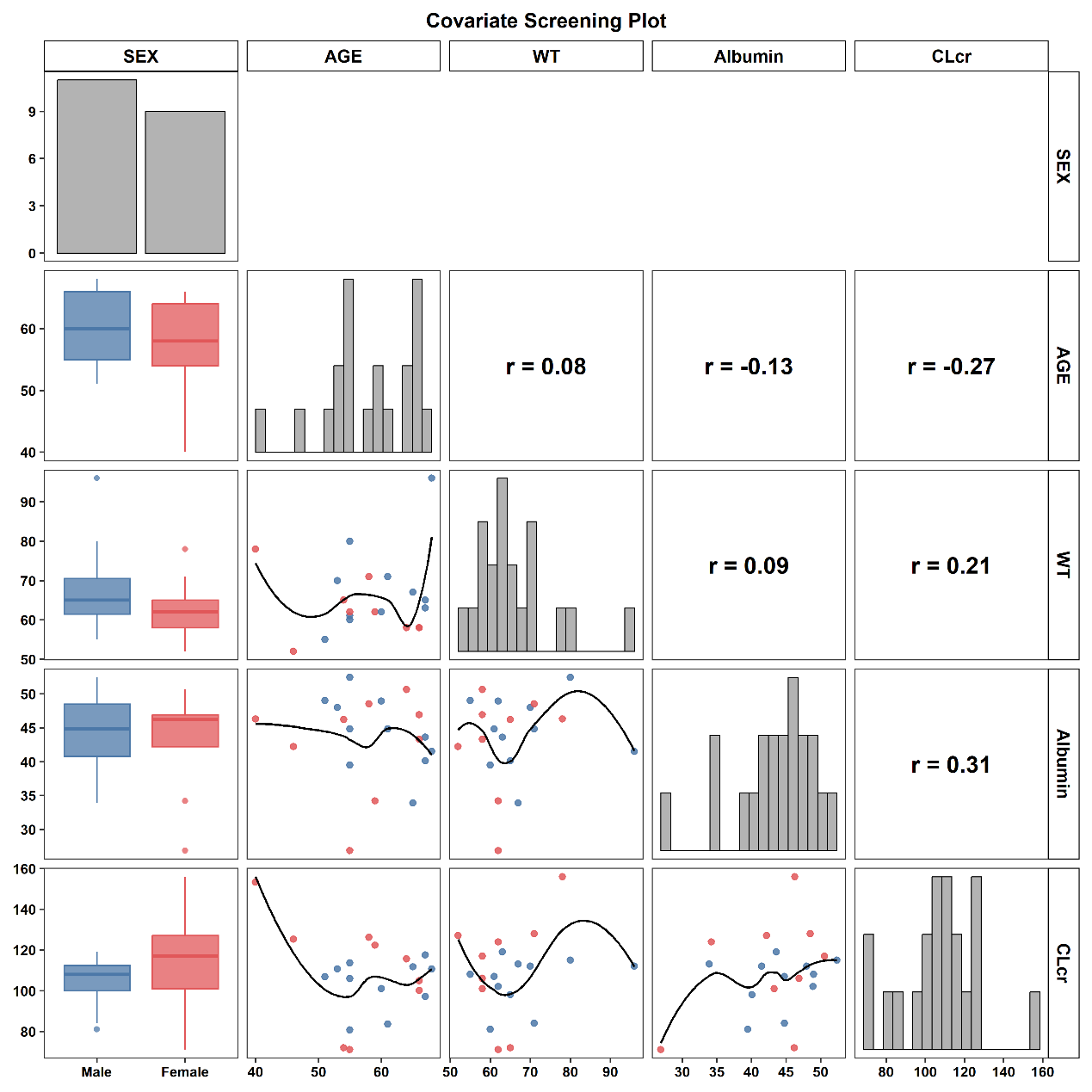


**Supplementary Figure 2.** Covariate screening plot showing pairwise relationships among covariates. Diagonal panels display variable distributions (histograms for continuous, bars for categorical). Lower panels show scatterplots or boxplots with LOESS smoothing, colored by SEX. Upper panels display Pearson correlation coefficients for continuous variables. This allows visual assessment of variable distributions, group differences, non-linear trends, and potential multicollinearity.


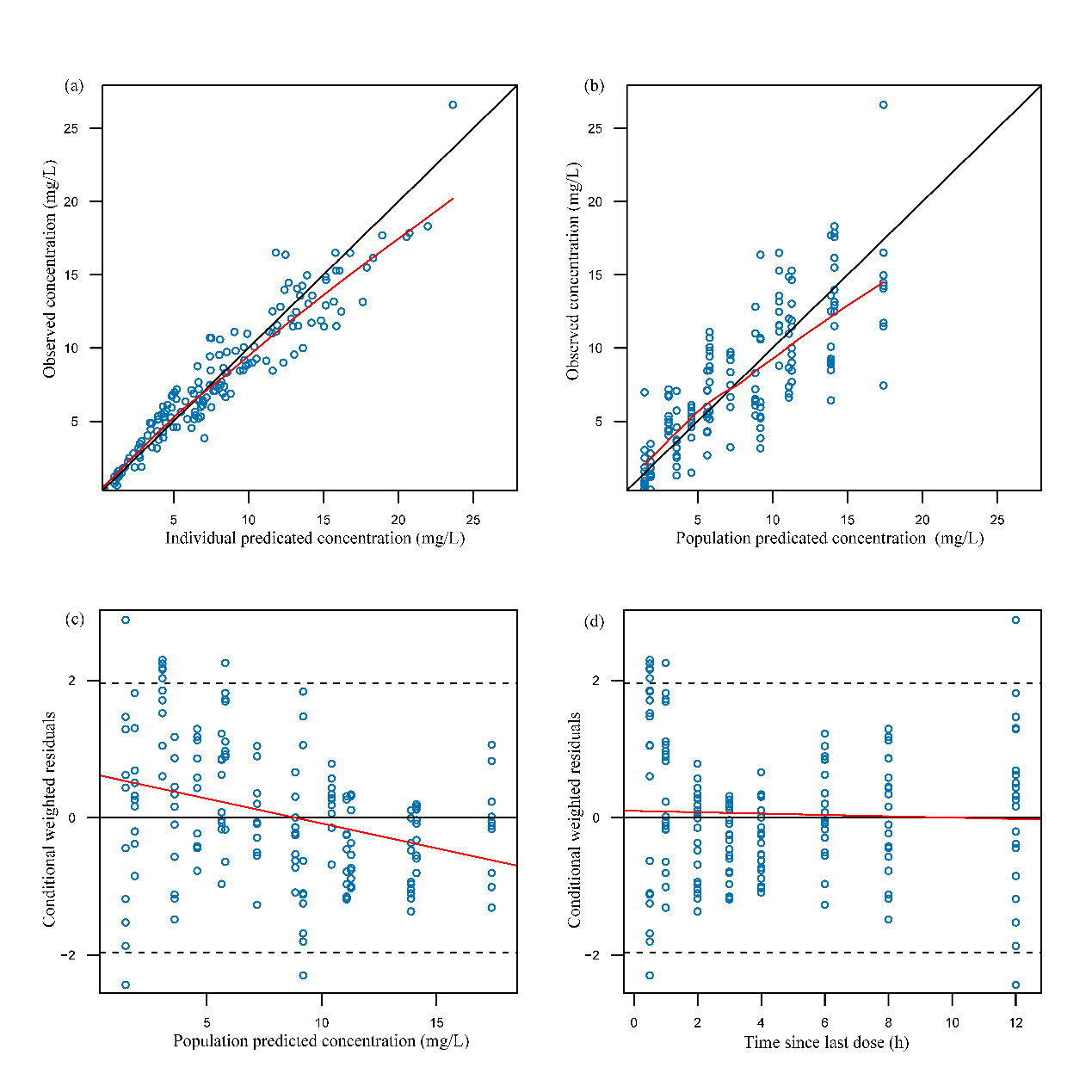


**Supplementary Figure 3.** Goodness-of fit plots obtained from the base linezolid population pharmacokinetic model. (a) Observation concentration vs individual predicated concentration, (b) Observation concentration vs population predicated concentration， (c) Conditional weighted residuals vs the population predictions, (d) Conditional weighted residuals vs the time after dosing. The red curves represent the locally weighted scatterplot smoothing lines.


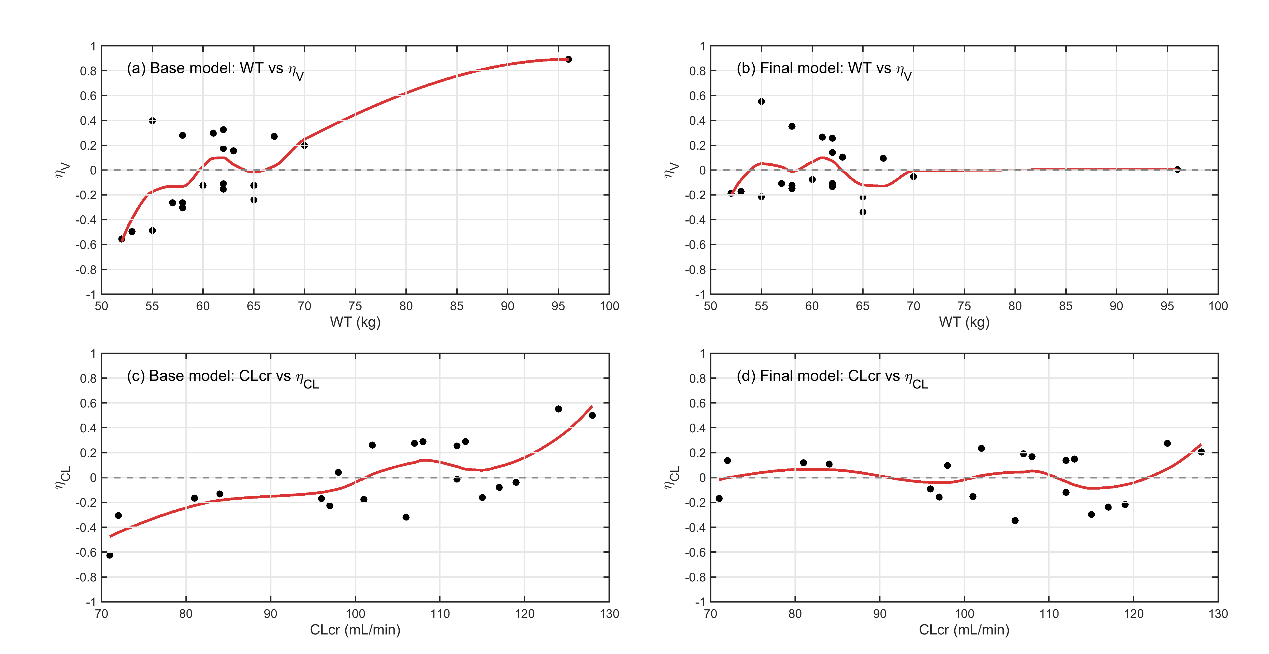


**Supplementary Figure 4.** Scatter plot of the correlation between inter-individual variations of linezolid pharmacokinetic parameters versus covariates. (a) The relationship between the inter-individual variation of distribution volume and body weight based on the basic model, (b) The relationship between the inter-individual variation of distribution volume and body weight based on the final model, (c) The relationship between the inter-individual variation of clearance and creatinine clearance based on the basic model, (d) The relationship between the inter-individual variation of clearance and creatinine clearance based on the final model. The red curves represent the Loess lines.


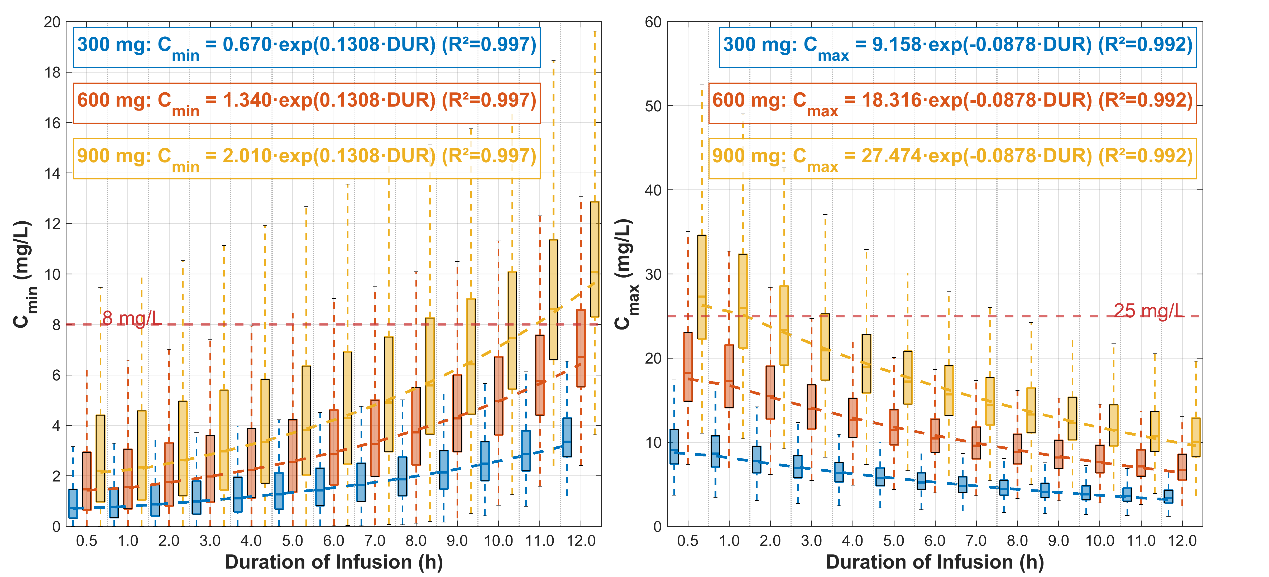


**Supplementary Figure 5.** The minimum and maximum values of the steady-state concentration with duration of infusion. The dotted lines represent the fitted curves.


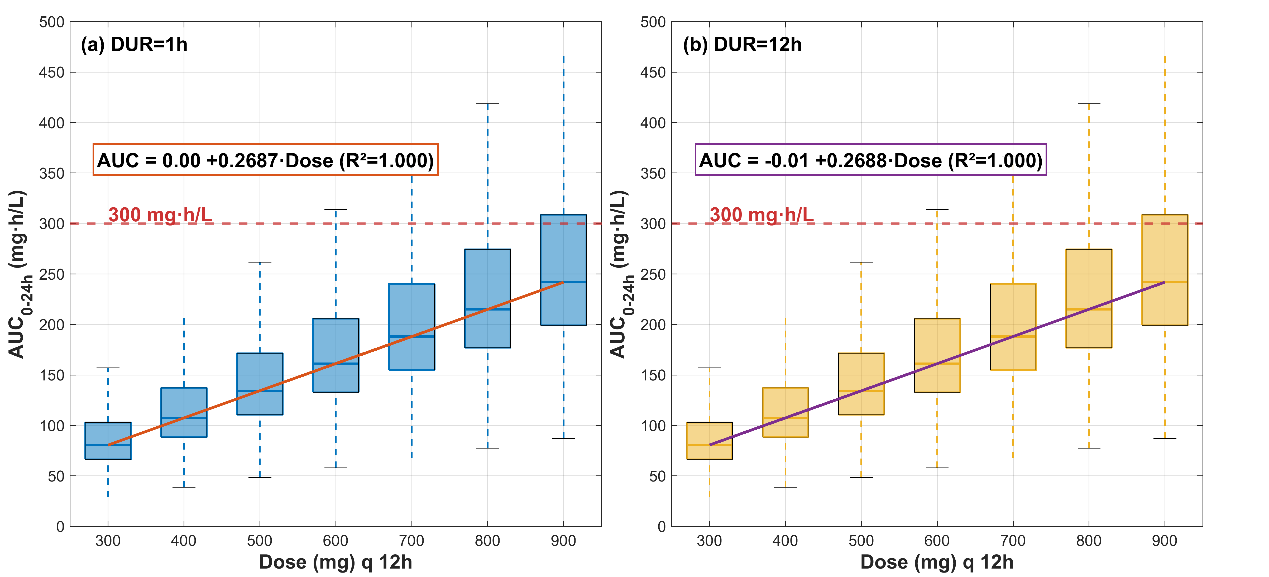


**Supplementary Figure 6.** The AUC_24_ of the steady-state concentration with dose. The lines represent the fitted curves.


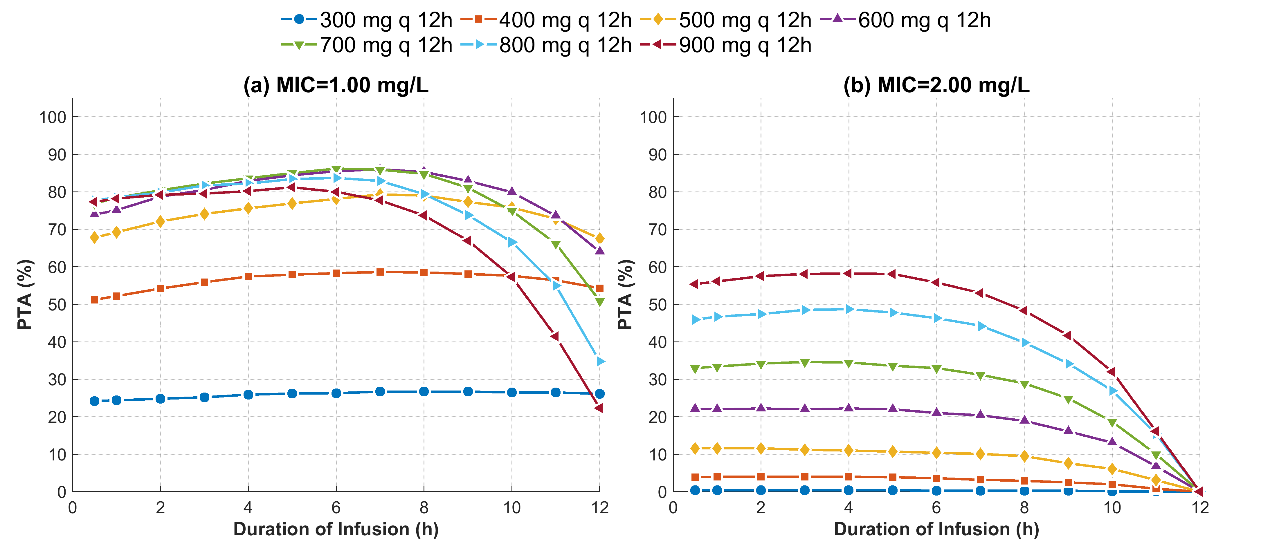


**Supplementary Figure 7.** PTA of various linezolid dosing regimens with duration of infusion for high MIC (mg/L).


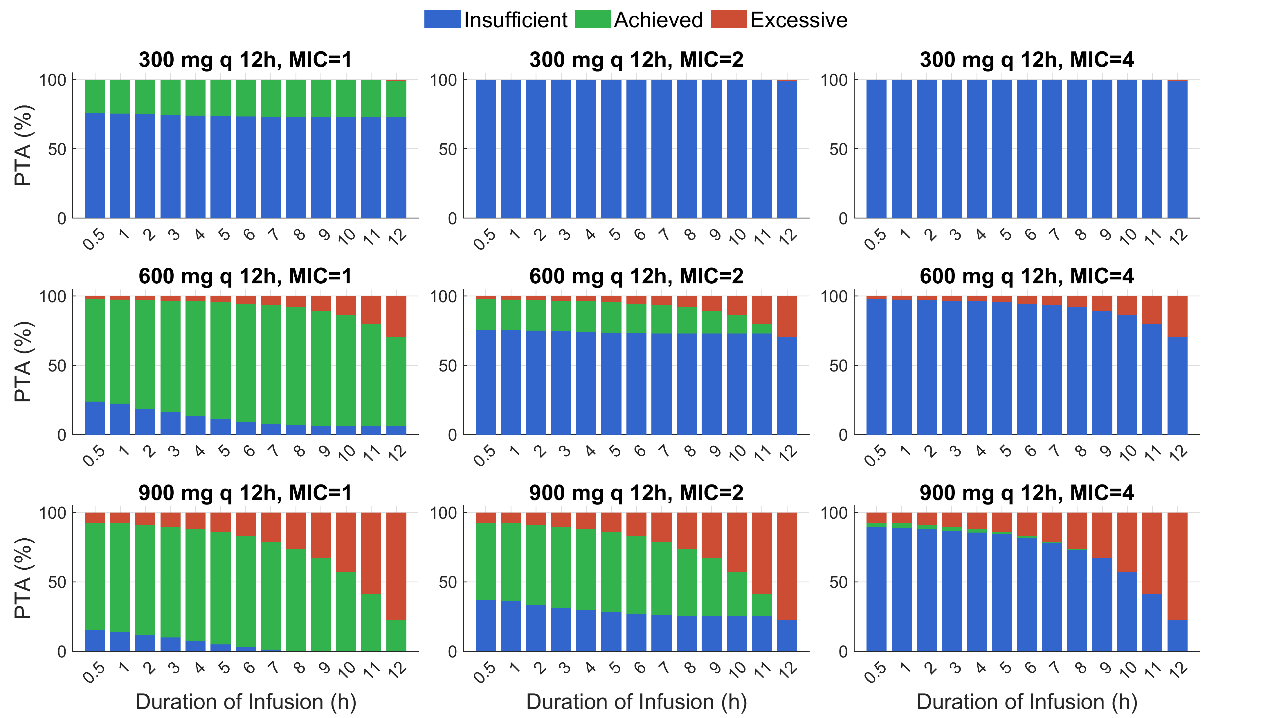


**Supplementary Figure 8.** Stacked bar graph of the PTA of various linezolid dosing regimens with duration of infusion for high MIC (mg/L).

## Supplementary Tables

**Supplementary Table 1.** Summary of covariate model building steps from base to final population-pharmacokinetic model of linezolid.

| Model No. | Model description | OFV | △OFV | P value | Acceptance/  Significance |
| --- | --- | --- | --- | --- | --- |
| Forward inclusion | |  |  |  |  |
| 1 | Base model | 380.62 |  |  |  |
| 2 | Add age on V | 379.275 | -1.345 | >0.05 | No |
| 3 | Add WT on V | 366.107 | -14.513 | <0.05 | Yes |
| 4 | Add Albumin on V | 380.594 | -0.026 | >0.05 | No |
| 5 | Add sex on V | 376.015 | -4.605 | <0.05 | Yes |
| 6 | Add CLcr on V | 379.326 | -1.294 | >0.05 | No |
| 7 | Add age on CL | 380.579 | -0.041 | >0.05 | No |
| 8 | Add WT on CL | 380.039 | -0.581 | >0.05 | No |
| 9 | Add Albumin on CL | 380.047 | -0.573 | >0.05 | No |
| 10 | Add sex on CL | 380.443 | -0.177 | >0.05 | No |
| 11 | Add CLcr on CL | 366.913 | -13.707 | <0.05 | Yes |
| 12 | Add sex on V to model #3 | 363.703 | -2.404 | >0.05 | No |
| 13 | Add CLcr on CL to model #3 | 352.546 | -13.561 | <0.05 | Yes |
| Backward elimination | |  |  |  |  |
| 14 | Remove CLcr on CL from model #13 | 366.107 | 13.561 | <0.01 | Yes |
| 15 | Remove WT on V from model #13 | 366.913 | 14.367 | <0.01 | Yes |

OFV: Objective function value; WT: Body weight; CLcr: Creatinine clearance.
